# Risk Factors of Preterm Birth in Nepal: A Hospital-Based Matched Case-Control Study

**DOI:** 10.3389/frph.2021.697419

**Published:** 2021-08-30

**Authors:** Richa Acharya, Pratik Khanal, Hari Krishna Bhattarai, Archana Amatya

**Affiliations:** ^1^Institute of Medicine, Tribhuvan University, Kathmandu, Nepal; ^2^Nepal Development Society, Bharatpur, Nepal

**Keywords:** preterm birth, risk factors, Nepal, delivery, newborn

## Abstract

**Background:** Preterm birth is a significant cause of neonatal death globally. Nepal is in the 20th position in the world, with the highest rate of preterm deliveries. The risk factors of preterm birth have not been fully identified and established in Nepal. The study aims to identify risk factors of preterm birth among women who underwent delivery in a tertiary maternal hospital in Nepal.

**Methods:** This study employed a hospital-based matched case-control study design. The case included women who delivered before 37 weeks of gestation, and women who delivered between 37 and 42 weeks of gestation served as controls. The ratio of the case to control was 1:2, and matching was done for the type of delivery. The first author collected the data in the Paropakar Maternity and Women's Hospital between December 2015 and January 2016. Face-to-face interviews were conducted using a structured questionnaire. Backward conditional logistic regression was performed to identify the independent risk factors of preterm birth.

**Results:** Antihelminthic treatment during pregnancy was found to be protective for preterm birth. Women performing intensive physical work during their pregnancy and women exposed to indoor air pollution were more likely to have a preterm birth than women not performing intensive physical work and women not exposed to indoor pollution, respectively.

**Conclusions:** Women who had not consumed antihelminthic drugs per protocol, those exposed to indoor air pollution, and those who performed intensive work during pregnancy were at higher risk for preterm birth. Maternal health programs can encourage women to consume antihelminthic drugs, take proper rest during pregnancy, and prevent indoor pollution exposure.

## Introduction

An estimated 15 million babies (more than 1 in 10 babies) are born preterm globally (1). Death of infants is common if they are preterm (2), and almost 1 million children die each year globally due to complications related to preterm birth (1). Preterm birth is any childbirth that occurs before 37 weeks of pregnancy or before 259 days of gestation since the first day of the woman's last menstrual period (3). Based on gestational age, preterm birth is categorized as extremely preterm occurring before 28 weeks; very preterm occurring at 28–31 weeks; moderate preterm occurring between 32 and 33 weeks; and late preterm occurring at 34 to <37 weeks of gestation (4). Preterm infants have higher rates of respiratory distress, apnea, temperature instability, seizures, hypoglycemia, and feeding difficulties. Many survivors suffer from neuro-developmental impairments, including cerebral palsy, intellectual disability, sensory impairments, and behavioral problems, including attention, visual processing, academic progress, and executive function (5).

The majority of preterm births (~85%) occur in Africa and the South-East Asia region (6). Nepal is a low-income South Asian country whose neonatal mortality rate (NMR) is 21 and infant mortality rate (IMR) is 32 per thousand live births according to the Nepal Demographic Health Survey (NDHS) 2016. Through the decades of investment in public health programs, maternal and child survival has improved over the years in the country. For instance, IMR has decreased from 78 deaths per thousand live births to 32 deaths in 2016 (7). However, Nepal has the 20th highest rate of preterm deliveries in the world (8), and there are no adequate data on prevalence and mortality associated with preterm birth (9); even if available, they are not collected and reported using standard definitions (10). In 2010, the worldwide estimates of preterm birth stated that 14 out of 100 babies born in Nepal were preterm resulting in around 1 million preterm births and 10,000 neonatal deaths due to complications of preterm birth (1). In 2015, the UNICEF report revealed that the leading cause of neonatal deaths in Nepal was prematurity (30.8 percent of the total neonatal deaths) (11).

Previous literature has identified a research gap in identifying risk factors of preterm birth in low-income countries, including Nepal (6, 12, 13). Therefore, it is crucial to systematically identify the risk factors of preterm birth among Nepalese women. In this context, this study aimed to determine the risk factors of preterm births among newly delivered women attending a tertiary-level maternity hospital in Nepal.

## Methods

### Study Design and Study Population

**Figure 1:**
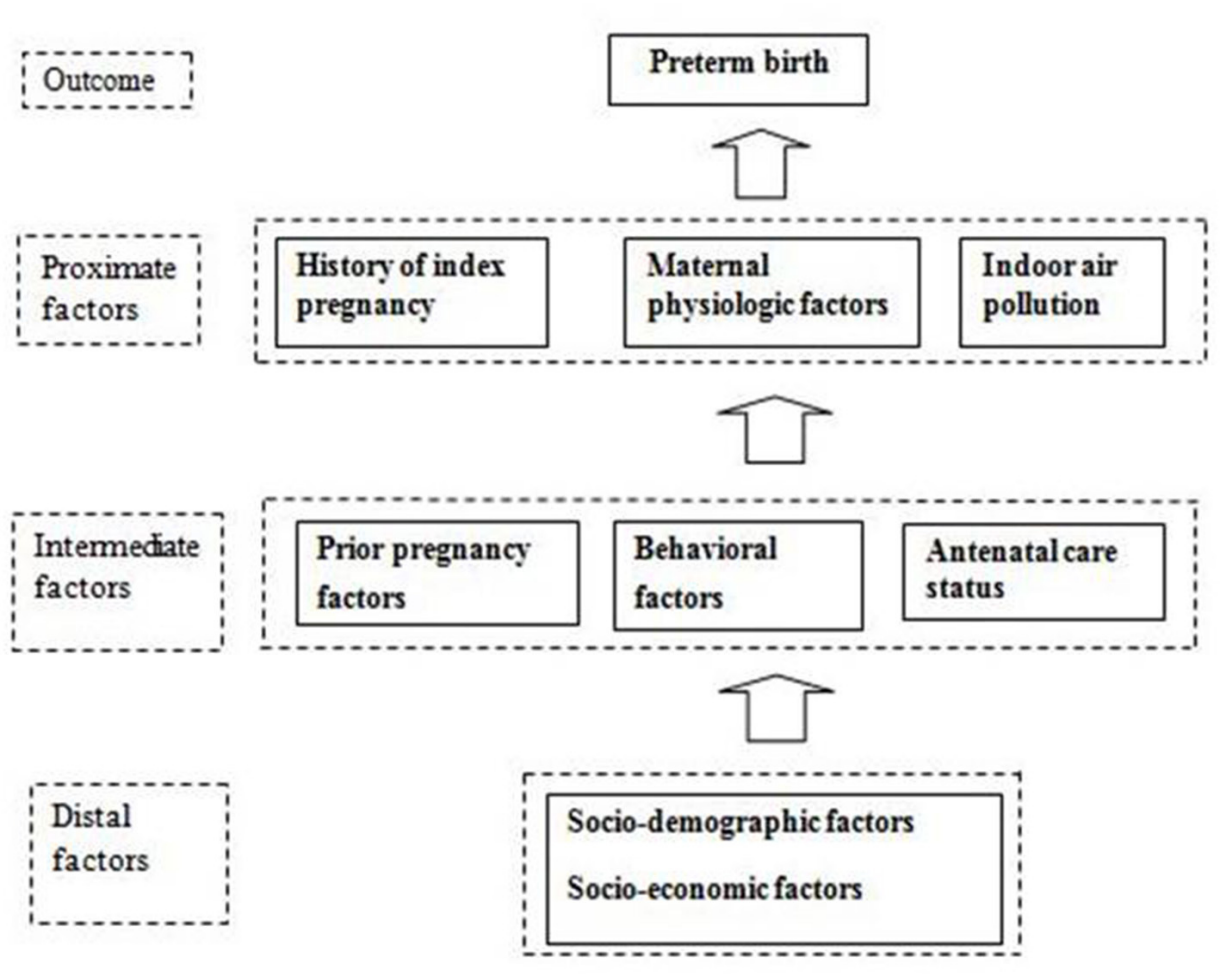
Conceptual framework of the study.

This study employed an institution-based retrospective case-control study design. The study participants were women of reproductive age of 15–49 years who had recently delivered a baby in the hospital. Cases were the women who delivered a single live newborn before 37 weeks of gestation. Controls were the women who delivered a single live newborn at or after 37 weeks and before 42 weeks of gestation, matched to the cases by type of delivery. This study was conducted in Paropakar Maternity and Women's Hospital (PMWH), located in Kathmandu, Nepal. The PMWH is the only tertiary-level public maternity hospital in the country. The conceptual framework of the study is shown in Figure 1. Data collection was done in December 2015 and January 2016. The study obtained ethical approval from the Institutional Review Committee of the Institute of Medicine, Tribhuvan University. Study participants provided written informed consent before the data collection.

### Sample Size Calculation and Sampling Technique

We calculated the sample size taking power at 80%, confidence level as 95%, a ratio of control to case as 2:1, an odds ratio of 3.23, and the percentage of controls exposed as 7.8 (14). Considering a 10% non-response rate, 94 cases and 188 controls were calculated as the final sample size for the study. We used the StatCalc application of Epi Info version 7 (Centers for Disease Control and Prevention, USA) to calculate the sample size.

**Figure 2:**
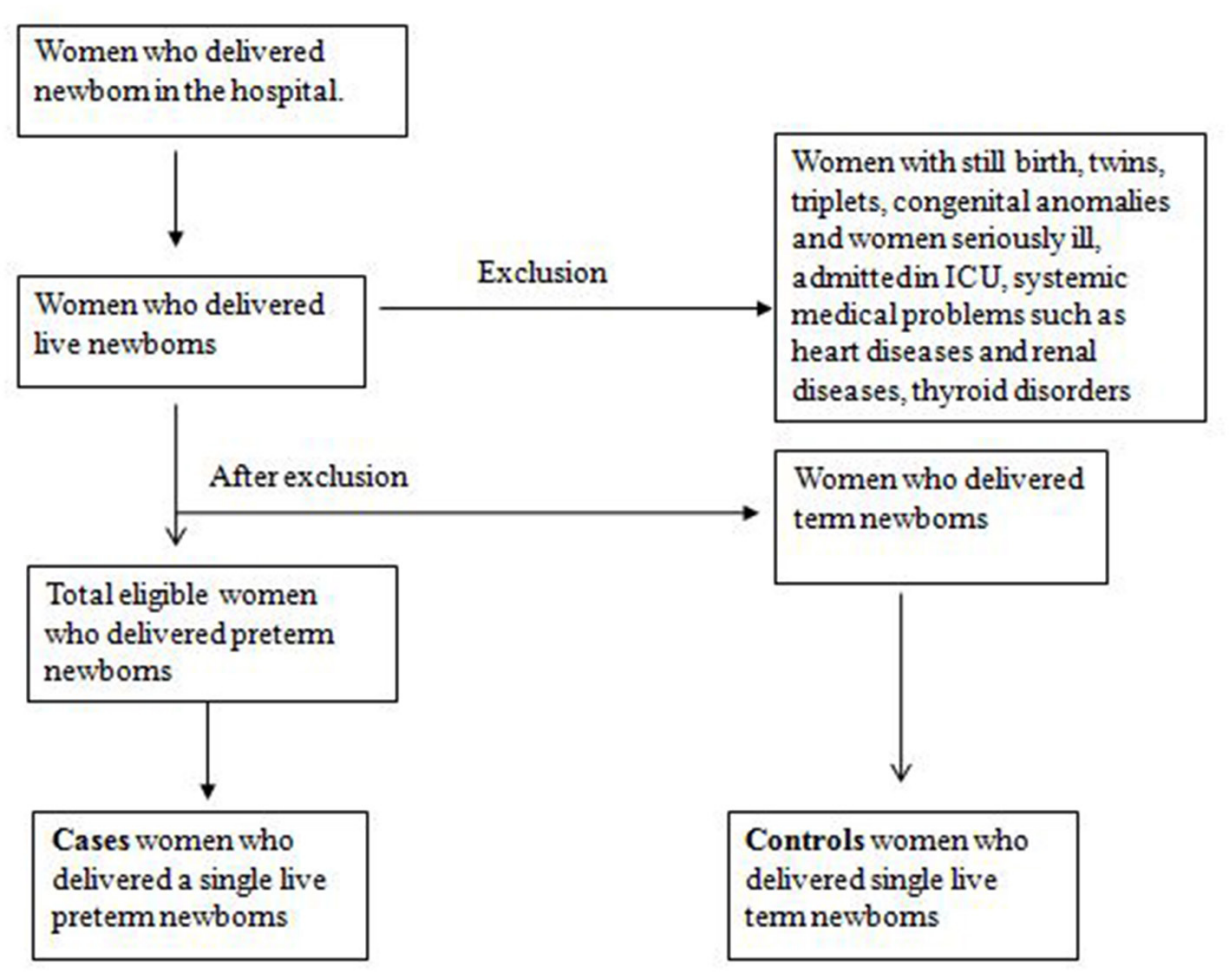
Sampling strategy.

The confinement books of the labor room and birthing center and in/out record book of the post-operative ward were checked daily to identify the birth of preterm newborns by vaginal delivery and the preterm newborns by lower section cesarean section (LSCS). The eligible controls matched to a case by type of delivery, delivering immediately after a case, were searched, and approached in postnatal ward A and ward B of the hospital and recruited for the study. The sampling strategy is presented in Figure 2. We selected two matched controls by type of delivery for every case. If the case delivered a preterm newborn by vaginal delivery, two controls who underwent vaginal delivery immediately after the case were selected. Similarly, if the case delivered preterm newborns by LSCS, we immediately selected two controls delivered by LSCS.

### Study Variables

The outcome variable of the study was preterm birth. Independent variables included socio-demographic and socio-economic factors, pregnancy-related factors, previous history and antenatal complications-related factors, nutritional status, and behavioral and environmental factors. The study variables are presented in Table 1. Similarly, operational definitions of some variables are described in Supplementary Material File 1.

**Table 1:** Study variables.

**S.N**.	**Variables**	**Categories**
**A**	**Dependent variable**	
1	Preterm birth	Yes, no
**B**	**Independent variables**	
**B1**	**Socio-demographic factors**	
1	Age of women at first pregnancy	<20 years, 20–24 years, 25–29 years, 30–34 years, ≥35 years
2	Age of women at current pregnancy	<20 years, 20–24 years, 25–29 years, 30–34 years, ≥35 years
3	Ethnicity	Dalits, disadvantaged Janajatis, disadvantaged non-Dalit Tarai caste, religious minorities, advantaged Janajatis, privileged caste
4	Religion	Hindu, Buddhist, Muslim, Christian, others
**B2**	**Socio-economic factors**	
1	Women's education	Illiterate, primary, lower secondary, secondary, higher secondary, university
2	Husband's education	Illiterate, primary, lower secondary, secondary, higher secondary, university
3	Women's occupation	Farmer, labor work/work in daily wage, housemaker, service, and business
4	Husband's occupation	Farmer, labor work/work in daily wage, service, business, and foreign employment
5	Wealth quintile	Lowest, second, middle, fourth, and highest
**B3**	**Pregnancy-related factors**	
1	Gravida	Primigravida, multigravida, grand multigravida
2	Parity	Primiparity, multiparity, grand multiparity
3	Birth interval	<2 years, more than or equal to 2 years
4	Initiation of ANC visits	Within 4 months, after 4 months
5	Frequency of ANC visits	<4 times, more than or equal to 4 times
6	Place of ANC visits	Hospitals, primary health care centers, health posts, private clinics
7	Iron consumption	No, regular, irregular
8	Calcium consumption	No, regular, irregular
9	TT/TD immunization	2 doses, 1 dose, no
10	Antihelminthic treatment	Yes, no
11	Use of other drugs during pregnancy	Yes, no
12	Intensive physical work performed during pregnancy	Yes, no
**B4**	**Previous history and pregnancy-related factors**	
1	Previous histories of preterm birth	Yes, no
2	Pregnancy complications	Yes, no
**B5**	**Nutritional status**	
1	Early pregnancy BMI	Underweight, normal, overweight/obese
2	Height of women	Normal (more than or equal to 145 cm), short (<145 cm)
3	Anemia	Yes, no
**B6**	**Behavioral and environmental factors**	
1	Tobacco smoking	Yes, no
2	Use of alcohol during pregnancy	Yes, no
3	Hours of sleep per 24 h	<7 h, 7–8 h, ≥9 h
4	Exposure to second-hand smoking	Yes, no
5	Exposure to indoor air pollution	Yes, no

### Operational Definition of the Variables

#### Iron Consumption

It was classified as no for women who did not consume iron tablets in their current pregnancy.

Iron consumption was marked as regular for women who started consuming iron at the start of the 4th month and were taking it daily, and irregular for women who did not take iron at the start of 4th month or women who were not consuming iron daily.

#### Calcium Consumption

Calcium consumption was classified as no for women who did not consume calcium tablets in their current pregnancy, regular for women who took calcium at the start of 4th month and were taking it daily, and irregular for women who did not take calcium at the start of 4th month or women who were not consuming it daily.

#### Drug Use

This was classified as the use of any kind of drug during pregnancy except folic acid, iron, calcium, and albendazole. It was dichotomized as yes or no.

#### Intensive Work Performed During Pregnancy

This was defined as any heavy work the women performed during pregnancy. This included fetching water with large buckets; lifting heavy loads; and washing clothes/utensils for long and labor-intensive work (construction work). For bivariate analysis, this variable was classified as performed intensive work and not performed intensive work.

#### Exposure to Indoor Air Pollution

It was adopted from the Nepal Demographic Health Survey 2011. Exposure to indoor air pollution was defined as women who reported that they cook inside the home using solid fuel (coal, lignite, charcoal, wood, and other traditional materials such as agricultural crop waste and animal dung).

### Data Collection Methods

Participants were invited to participate in a face-to-face interview using a structured questionnaire which took around 30 min. Information was also abstracted referring to the ultrasonography (USG) report for the period of gestation, antenatal care (ANC) card for antenatal, medical, and reproductive histories, prenatal records for complications during pregnancy, and postnatal medical records for conditions of newborns. We adapted the study tool from NDHS, 2011 (15).

### Data Management and Analysis

Collected data were entered in EpiData version 3.1 and analyzed using IBM Statistical Package for Social Science (SPSS) version 20 (IBM SPSS Statistics, USA). After importing data from EpiData, we conducted data checking, cleaning, editing, and recoding before analysis. We then conducted descriptive analysis in terms of frequency tables by splitting cases and controls. Frequency and percentages were calculated for the categorical variables and mean, and standard deviation (SD) was calculated for continuous variables. Chi-square test was performed to determine the difference in the exposure between cases and controls. Those variables with *p*-value < 0.1 were then fitted into the multivariable logistic regression model to identify the predictors of pre-term birth.

Backward conditional logistic regression analysis was performed and the probability of removal was set at *p* > 0.1. We carried out Hosmer and Lemeshow goodness of fit to ensure that the model was fit. The analysis was fitted in the backward logistic regression model, logit P (X) = β_0_+ β_1_X_1_ + β_2_X_2_ + β_3_X_3_ + β_4_X_4_ + …………. + β_i_Xi, where P (X) is the log odds of the outcome variable, β_0_ is constant, βi is the regression coefficient, and Xi is the exposure variable.

Before fitting into the multivariable regression model, we checked the multi-collinearity of the variables, and the maximum variance inflation factor (VIF) obtained were 29.76 and 13.97 for birth spacing and parity, respectively. After removing the variable “birth spacing” from the model, the maximum VIF was 8.79 for parity, which was fitted in the model.

A total of 14 variables showed statistical significance in the bivariate analysis: Age of women at current pregnancy, educational status of women, gravida, parity, calcium consumption, antihelminthic treatment, use of drugs in pregnancy, intensive work during pregnancy, early pregnancy BMI, history of preterm birth, pregnancy-related problems, sleep hours per 24 h, exposure to second-hand smoking, and exposure to indoor air pollution. We fitted 13 variables into the multivariable regression model. Variable “history of preterm birth” was removed due to insufficient data in the control group.

## Results

The proportions of extremely preterm (<28 weeks), very preterm (28– <32 weeks), moderate preterm (32– <34weeks), and late preterm (34– <37) in this study were 5.3, 12.8, 10.6, and 71.3%, respectively.

### Socio-Demographic and Socio-Economic Characteristics

The mean age of study participants at their first pregnancy and current pregnancy were 21 and 24 years, respectively for both case and control groups. The majority (82.3%) of the study participants were Hindu. More than one-third of the study participants (37.6%) were from the disadvantaged *Janajatis* ethnic group with almost equal proportions in both case and control groups. Two out of five participants had completed secondary level education with similar proportions in cases and controls. Nearly two-thirds of the participants (64.5%) were homemakers with a slightly higher proportion in the control (66%) group than in the case (61.7%) group. One out of five study participants were in the lowest wealth quintile with more controls (21.3%) in the lowest wealth quintile than cases (17.0%) (Table 2).

**Table 2:** Socio-demographic and economic characteristics of the study participants.

**Variables**	**Study group**		**Total**
	**Case (*n* = 94) *n* (%)**	**Control (*n* = 188)*n* (%)**	**(*n* = 282)*n* (%)**
**Socio-demographic characteristics**			
**Age of women at first pregnancy (years)**			
<20	37 (39.4)	65 (34.6)	102 (36.2)
20–24	39 (41.5)	83 (44.1)	122 (43.3)
25–29	13 (13.8)	36 (19.1)	49 (17.4)
30–34	5 (5.3)	4 (2.1)	9 (3.1)
**Mean age at first pregnancy (years** **±SD)**	21.26 ± 4.04	21.44 ± 3.70	21.38 ± 3.81
**Age of women at current pregnancy (years)**			
<20	26 (27.7)	29 (15.4)	55 (19.5)
20–24	28 (29.8)	72 (38.3)	100 (35.5)
25–29	22 (23.4)	63 (33.5)	85 (30.1)
30–34	16 (17.0)	20 (10.6)	36 (12.8)
35–39	2 (2.1)	4 (2.1)	6 (2.1)
**Mean age at current pregnancy (years** **±SD)**	24.09 ± 5.42	24.27 ± 4.35	24.21 ± 4.73
**Ethnicity**			
Dalits	8 (8.5)	16 (8.5)	24 (8.6)
Disadvantaged Janajatis	36 (38.3)	70 (37.2)	106 (37.6)
Disadvantaged Non-Dalit Tarai people	5 (05.3)	3 (1.6)	8 (2.8)
Advantaged Janajatis	12 (12.8)	35 (18.6)	47 (16.6)
Upper caste	33 (35.1)	64 (34.0)	97 (34.4)
**Religion**			
Hindu	80 (85.1)	152 (80.9)	232 (82.3)
Buddhist	10 (10.6)	25 (13.3)	35 (12.4)
Christian	4 (4.3)	13 (05.9)	15 (5.4)
**Residence**			
Kathmandu Valley	21 (22.3)	41 (21.8)	63 (22.0)
Out of Kathmandu Valley	72 (77.7)	146 (78.2)	218 (78.0)
**Education of women**			
Illiterate	10 (10.6)	17 (09.0)	27 (09.6)
Literate	3 (3.2)	4 (2.1)	7 (2.5)
Primary	20 (21.3)	27 (14.4)	47 (16.7)
Secondary	39 (41.5)	75 (39.9)	114 (40.4)
Higher Secondary	11 (11.7)	52 (27.7)	63 (22.3)
University	11 (11.7)	13 (6.9)	24 (8.5)
**Education of husband**			
Illiterate	9 (9.6)	13 (7.0)	22 (7.9)
Literate	3 (3.2)	5 (2.7)	8 (2.9)
Primary	14 (14.9)	28 (15.1)	42 (15.0)
Secondary	35 (37.2)	83 (44.6)	118 (42.1)
Higher secondary	17 (18.1)	36 (19.4)	53 (18.9)
University	16 (17.0)	21 (11.3)	37 (13.2)
**Occupation of women**			
Farmer	9 (9.6)	26 (13.8)	35 (12.4)
Labor/work in daily wage	7 (7.4)	8 (4.3)	15 (5.3)
Homemaker/manager	58 (61.7)	124 (66.0)	182 (64.5)
Service	15 (16.0)	20 (10.6)	35 (12.4)
Business	5 (05.3)	10 (5.3)	15 (5.3)
**Occupation of husband**			
Farmer	9 (9.6)	10 (5.3)	19 (6.7)
Labor/work in daily wage	22 (23.4)	45 (23.9)	67 (23.8)
Service	34 (36.2)	74 (39.4)	108 (38.3)
Business	20 (21.3)	40 (21.3)	60 (21.3)
Foreign employment	9 (9.6)	19 (10.1)	28 (9.9)
**Wealth quintile**			
Lowest quintile	16 (17.0)	40 (21.3)	56 (19.9)
Second quintile	15 (16.0)	42 (22.3)	57 (20.2)
Middle quintile	18 (19.1)	38 (20.2)	56 (19.9)
Fourth quintile	25 (26.6)	32 (17.0)	57 (20.2)
Highest quintile	20 (21.3)	36 (19.1)	56 (19.9)

### Pregnancy-Related Characteristics

Among the case group women, 59.6% were primigravida, 36.2% were multigravida, and 4.3% were grand multigravida, while in the control group women, 47.3% were primigravida, 52.1% were multigravida, and 0.5% were grand multigravida. In the case group, 69.1% of women were primiparous, 29.8% were multiparous, and 1.1% were grand multiparous, while in the control group, 55.9% of women were primiparous and 44.1% were multiparous. Among multiparous and grand multiparous women, 12.5% of the case group and 3.7% of the control group reported <2 years of birth interval. Regarding pregnancy care, 31.9% of case group women and 11.2% of control group women had ANC check-ups <4 times. Likewise, 77.7% of case group women and 81.4% of control group women received ANC check-ups from doctors. The proportion of women who consumed iron regularly was 27.7% in the case group and 36.4% in the control group. Similarly, the proportion of women who consumed calcium regularly was 21.3% in the case group and 34.6% in the control group. Also, 8.5% of case group women were not immunized with tetanus toxoid (TT)/tetanus-diphtheria (TD) vaccine compared with 2.1% of control group women; and 64.9% of the case group women and 46.8% of the control group women did not have antihelminthic treatment. More women in the case group (11.7%) than those in the control group (2.1%) performed intensive work during pregnancy (Table 3).

**Table 3:** Pregnancy-related characteristics of the study participants.

**Variables**	**Study group**		**Total**
	**Case (*n* = 94) *n* (%)**	**Control (*n* = 188)*n* (%)**	**(*n* = 282)**
**Gravida**			
Primigravida	56 (59.6)	89 (47.3)	145 (51.4)
Multigravida (2–4)	34 (36.2)	98 (52.1)	132 (46.8)
Grand multigravida (≥5)	4 (4.2)	1 (0.5)	5 (1.8)
**Parity**			
Primiparous	65 (69.1)	105 (55.9)	170 (60.3)
Multiparous	28 (29.8)	83 (44.1)	111 (39.4)
Grand multiparous	1 (1.1)	-	1 (0.3)
**Birth interval (*****n*** **=** **114)**			
<2 years	4 (12.5)	3 (3.7)	8 (7.0)
2 years or more	28 (87.5)	79 (96.3)	107 (93.9)
**ANC visits**			
<4 times	30 (31.9)	21 (11.2)	51 (18.1)
≥4 times	64 (68.1)	167 (88.8)	231 (81.9)
**Place of ANC visits**			
Hospital	65 (69.1)	144 (76.6)	209 (74.1)
Primary health care centers	7 (7.4)	12 (6.4)	19 (6.7)
Health posts	14 (14.9)	24 (12.8)	38 (13.5)
Private clinics	8 (8.5)	8 (4.3)	16 (5.7)
**Antenatal care providers**			
Doctor	73 (77.7)	153 (81.4)	226 (80.1)
Nurse	17 (18.1)	31 (16.5)	48 (17.0)
Paramedics	3 (3.2)	3 (1.6)	6 (2.1)
MCHW	1 (1.1)	1 (0.5)	2 (0.7)
**Iron consumption**			
Regular	26 (27.7)	68 (36.4)	94 (33.3)
Irregular	60 (63.8)	104 (55.6)	164 (58.2)
No	8 (8.5)	16 (8.5)	24 (8.5)
**Calcium consumption**			
Regular	20 (21.3)	65 (34.6)	175 (62.1)
Irregular	65 (69.1)	110 (58.5)	85 (30.1)
No	9 (9.6)	13 (6.9)	22 (7.8)
**TT/TD immunization**			
2 doses	75 (79.8)	173 (92.0)	248 (87.9)
1 dose	11 (11.7)	11 (5.9)	22 (7.8)
No	8 (8.5)	4 (2.1)	12 (4.3)
**Antihelminthic treatment**			
Yes	33 (35.1)	100 (53.2)	133 (47.2)
No	61 (64.9)	88 (46.8)	149 (52.8)
**Illicit use of drugs during pregnancy**			
Yes	15 (8.0)	16 (17.0)	31 (11.0)
No	173 (92.0)	78 (83.0)	251 (89.0)
**Intensive work performed during pregnancy**			
Performed	11 (11.7)	4 (2.1)	15 (5.3)
Not performed	83 (88.3)	184 (97.9)	267 (94.7)

### Previous Histories and Antenatal Complication-Related Characteristics

Among study participants, 2.1% of women in the case group and 1.1% in the control group had a previous history of stillbirth; 10.6% of the case group and 8.5% of the control group women had a history of spontaneous abortion, and 9.6% of the women in the case group and 9% of the women in the control group had a history of induced abortion. Similarly, 6.4% of the women in the case group had a history of preterm birth compared to 0.5% in the control group. Also, 1.1% of the case group women and 1.6% of the control group had a history of delivering a baby with low birth weight.

The proportion of women who had antenatal complications was 45.7% in the case group and 30.9% among control group women. Likewise, 7.4% of the case group women had vaginal bleeding, while no women in the control group had vaginal bleeding. A similar finding was reported in the case of eclampsia, where 4.3% of the case group women had eclampsia, with none had it in the control group. The proportion of women who had severe gestational hypertension was 2.1% in the case group women and 0.5% in the control group. An almost equal proportion of the case group women (1.1%) and control group women (1.6%) had oligohydramnios while 6.4% of the case group women and 3.2% of the control had a urine infection during their pregnancy (Table 4).

**Table 4:** Previous histories and antenatal complication-related characteristics of the study participants.

**Variables**	**Study group**		**Total**
	**Case (*n* = 94) *n* (%)**	**Control (*n* = 188)*n* (%)**	**(*n* = 282)**
**Previous history**			
**Stillbirth**			
Yes	2 (2.1)	2 (1.1)	4 (1.4)
No	92 (97.9)	186 (98.9)	278 (98.6)
**Spontaneous abortion**			
Yes	10 (10.6)	16 (8.5)	26 (9.2)
No	84 (89.4)	172 (91.5)	256 (90.8)
**Induced abortion**			
Yes	9 (9.6)	17 (9.0)	26 (9.2)
No	85 (90.4)	171 (91.0)	256 (90.8)
**Preterm birth**			
Yes	6 (6.4)	1 (0.5)	7 (2.5)
No	88 (93.6)	187 (99.5)	275 (97.5)
**Low birth weight**			
Yes	1 (1.1)	3 (1.6)	4 (1.4)
No	93 (98.9)	185 (98.4)	278 (98.6)
**Pregnancy complications**			
Yes	43 (45.7)	58 (30.9)	101 (35.8)
No	51 (54.3)	130 (69.1)	181 (64.2)
**Vaginal bleeding**			
Yes	7 (7.4)	0 (0)	7 (2.5)
No	87 (92.6)	188 (100)	275 (97.5)
**Severe gestational hypertension**			
Yes	2 (2.1)	1 (0.5)	3 (1.1)
No	92 (97.9)	187 (99.5)	279 (98.9)
**Gestational hypertension**			
Yes	6 (6.4)	14 (7.4)	20 (7.1)
No	88 (93.6)	174 (92.6)	262 (92.9)
**Eclampsia**			
Yes	4 (4.3)	0 (0)	4 (1.4)
No	90 (95.7)	188 (100)	278 (98.6)
**Oligohydramnios**			
Yes	1 (1.1)	3 (1.6)	4 (1.4)
No	93 (98.9)	185 (98.4)	278 (98.6)
**Hepatitis B positive**			
Yes	0 (0)	1 (0.5)	1 (0.4)
No	94 (100)	187 (99.5)	281 (99.6)
**Urine infection**			
Yes	6 (6.4)	6 (3.2)	12 (4.3)
No	88 (93.6)	182 (96.8)	270 (95.7)

### Nutritional Status

Among women in the case group, 24.5% were underweight and 12.8% were obese compared to 11.7% underweight and 16.5% obese in the control group women. More women in the control group (7.4%) had a short stature than those in the case group (4.3%). Women in the case group women (24.5%) had a higher proportion of anemia than those in the control group (22.3%) (Table 5).

**Table 5:** Nutritional status of the study participants.

**Variables**	**Study group**		**Total**
	**Case (*n* = 94) *n* (%)**	**Control (*n* = 188)*n* (%)**	**(*n* = 282)**
**Early pregnancy BMI**			
Underweight (BMI <18.5)	23 (24.5)	22 (11.7)	45 (16.0)
Normal (BMI 18.5–24.9)	59 (62.8)	135 (71.8)	194 (68.8)
Overweight/obese (BMI≥25)	12 (12.8)	31 (16.5)	43 (15.2)
**Height of women**			
Normal stature (>145 cm)	90 (95.7)	174 (92.6)	264 (93.6)
Short stature (<145 cm)	4 (4.3)	14 (7.4)	18 (6.4)
**Anemia**			
No	71 (75.5)	146 (77.7)	217 (77.0)
Yes	23 (24.5)	42 (22.3)	65 (23.0)
**Type of anemia**			
Mild	14 (60.9)	33 (78.6)	47 (72.3)
Moderate	9 (39.1)	9 (21.4)	18 (27.7)

### Behavioral and Environmental Characteristics

Among study participants, 86.2% in the case group and 93.1% in the control group had no smoking history. Among those who smoked, 3.2% of the case and 1.1% of the control group women smoked 4–5 cigarettes per day. One out of five women in the case group and one in 10 in the control group had a history of drinking alcohol during pregnancy. A nearly equal proportion of the case (2.1%) and control group women (1.1%) had <6 h of sleep.

A higher proportion of the case group (53.2%) was exposed to second-hand smoking than the control group (33%). Similarly, 36.2% of the case group and 16% of the control group were exposed to indoor air pollution (Table 6).

**Table 6:** Behavioral and environmental characteristics among the study participants.

**Variables**	**Study group**		**Total**
	**Case (*n* = 94)*n* (%)**	**Control (*n* = 188)*n* (%)**	**(*n* = 282)**
**Behavioral factors**			
**Tobacco smoking**			
Never smoked	81 (86.2)	175 (93.1)	256 (90.8)
Smoked but not during pregnancy	6 (6.4)	5 (2.7)	11 (3.9)
Smoked 1–3 cigarettes per day	4 (4.3)	6 (3.2)	10 (3.5)
Smoked 4–5 cigarettes per day	3 (3.2)	2 (1.1)	5 (1.8)
**Use of alcohol during pregnancy**			
Yes	14 (19.5)	17 (9.0)	31 (110)
No	80 (85.1)	171 (91.0)	251 (89.0)
**Hours of sleep per 24 h**			
≤ 6	2 (2.1)	2 (1.1)	4 (1.4)
7–8	15 (16.0)	58 (30.9)	73 (25.9)
≥ 9	77 (81.9)	128 (68.1)	205 (72.7)
**Environmental factors**			
**Exposure to second-hand smoking**			
Yes	50 (53.2)	62 (33.0)	112 (39.7)
No	44 (46.8)	126 (67.0)	170 (60.3)
**Exposure to indoor air pollution**			
Yes	34 (36.2)	30 (16.0)	64 (22.7)
No	60 (63.8)	158 (84.0)	218 (77.3)

### Risk Factors for Preterm Birth

In adjusted analysis, antihelminthic treatment, intensive work performed during pregnancy, and exposure to indoor air pollution were identified as independent risk factors of preterm birth.

Those women who did not receive antihelminthic treatment had higher odds for preterm birth (AOR: 2.19, 95% CI: 1.21–3.93) than women who had antihelminthic treatment. Similarly, women who performed intensive work during their pregnancy were five times more likely to have a preterm birth than those who did not do intensive work (AOR: 5.37, 95% CI:1.39–20.68). In addition, exposure to indoor air pollution increased the risk of preterm birth (AOR: 2.95, 95% CI: 1.50–5.79).

Based on the findings, the final equation can be derived as

Preterm birth = −3.122 (constant) + 0.737 × X_1_ + 1.643 × X_2_ ++ 1.120 × X_3_

Where, X_1_ = antihelminthic treatment; X_2_ = intensive work performed during pregnancy; and X_3_ = exposure to indoor air pollution.

The final equation had a Nagelkerke *R*^2^ value of 0.325, showing that a 33% change in the outcome variable (preterm birth) was explained by exposure variables, i.e., no antihelminthic treatment during pregnancy, intensive work performed during pregnancy, and exposure to indoor air pollution. Hosmer and Lemeshow's goodness of fit was not significant (*p* = 0.221), indicating that the model fits the data (Table 7).

**Table 7:** Predictors of preterm birth.

**Variables**	**Preterm birth(*n* = 94)*N* (%)**	**Crude OR(95% CI)**	**Adjusted OR (95% CI)**
**Antihelminthic treatment**			
Yes	33 (35.1)	Ref.	Ref
No	61 (64.9)	2.1 (1.26–3.50)	2.19 (1.21–3.93)
**Intensive physical work**			
Yes	11 (11.7)	6.10 (1.89–19.71)	5.37 (1.39–20.68)
No	83 (88.3)	Ref.	Ref.
**Exposure to indoor air pollution**			
Yes	60 (63.8)	2.98 (1.68–5.29)	2.95 (1.50–5.79)
No	34 (36.1)	Ref.	Ref.
**Pregnancy-related problems**			
Yes	43 (45.7)	1.89 (1.13–3.15)	1.66 (0.91–3.01)
No	51 (54.3)	Ref.	Ref.
**Age of women at current pregnancy**			
<20 **y**ears	26 (27.7)	2.09 (1.15–3.82)	1.18 (0.57–2.45)
≥20 **y**ears	68 (72.3)	Ref.	Ref.
**Education of women**			
≤ class 8	52 (55.3)	1.67 (1.02–2.75)	1.46 (0.78–2.70)
>class 8	42 (44.7)	Ref.	Ref.
**Gravida**			
Primigravida and grand multigravida	60 (63.8)	1.92 (1.16–3.20)	1.13 (0.44–2.90)
Multigravida (2–4)	34 (36.2)	Ref.	Ref.
**Parity**			
Primiparous and grand multiparous	66 (70.2)	1.86 (1.09–3.16)	2.13 (0.77–5.88)
Multiparous	28 (29.8)	Ref	Ref.
**Calcium consumption**			
No/irregular	74 (78.7)	1.96 (1.09–3.49)	1.39 (0.72–2.68)
Regular	20 (21.3)	Ref.	Ref.
**Use of drugs during pregnancy**			
Yes	16 (17)	2.37 (1.11–5.03)	1.94 (0.79–4.78)
No	78 (83)	Ref.	Ref.
**Early pregnancy BMI**			
Underweight	23 (24.5)	2.40 (1.24–4.63)	1.59(0.75–3.36)
Overweight/obese	12 (12.8)	0.89 (0.43–1.84)	1.83 (0.46–2.54)
Normal	59 (62.8)	Ref.	Ref.
**Hours of sleep per 24 h**			
≤ 6 (Short sleep hours)	2 (2.1)	3.87 (0.50–29.75)	1.82 (0.16–20.22)
≥9 (Long sleep hours)	77 (81.9)	2.33 (1.23–4.39)	1.85 (0.89–3.83)
7–8 (Normal sleep hours)	15 (16.0)	Ref.	Ref.
**Exposure to second-hand smoking**			
Yes	50 (53.2)	2.31 (1.39–3.83)	1.61 (0.89–2.93)
No	44 (46.8)	Ref.	Ref.

*Ref: Reference category*.

## Discussion

This study identified risk factors of preterm birth among newborns in Nepal by using a case-control study design. Women who did not receive antihelminthic treatment during pregnancy, those exposed to indoor air pollution, and those who performed intensive work during pregnancy were identified as risk factors of preterm birth on our study of Nepalese women delivering in the tertiary hospital of Nepal.

The finding that women who did not have antihelminthic treatment were at higher risk of preterm birth was contrary to the Cochrane review (16) and Canadian study (17), which showed no association between antihelminthic treatment and preterm birth. However, very little information is available on the effect of antihelminthic treatment on preterm birth (18). Further research might be helpful to understand the relationship between antihelminthic treatment and preterm birth.

The risk of preterm birth was higher in women exposed to solid fuel while cooking. Our findings are consistent with the multicentric observational study done in Nepal (19) and other studies that provide evidence of solid fuels for cooking and increased risk of preterm birth (19, 20, 20–22). The combustion of biomass fuel emits high concentrations of airborne particulate matter and toxic chemicals, including carbon monoxide (CO), nitrogen dioxide (NO_2_), and sulfur dioxide (SO_2_). The oxygen content of maternal blood gets reduced when these pollutants are absorbed into the maternal bloodstream. Subsequently, oxygen delivery to the placenta is reduced, resulting in preterm delivery. It is biologically plausible that exposure to solid fuel smoke increases the risk of preterm birth (21). However, our findings and those of Liu et al. differ. The study done by Liu et al. in China showed no significant associations between solid fuels for cooking and preterm birth (23).

Our study showed that those women who performed intensive physical activity during their pregnancy had a significant association with the risk of preterm birth. A previous systematic review and meta-analysis showed that physically demanding work is associated with an increased risk of preterm birth (24). Studies done in Italy (25) and Iran (26) also found an increased risk of preterm birth in women employed in heavy work (25).

In our study, age of the women, ethnicity, religion, education, occupation, wealth quintile, gravid, parity, ANC visits, iron consumption, calcium consumption, TT/TD immunization, pregnancy-related problems, use of drugs during pregnancy, early pregnancy BMI, the height of women, anemia during pregnancy, past pregnancy-related characteristics, smoking, alcohol intake during pregnancy, sleep hours, and exposure to second-hand smoking did not show any significant association with preterm birth. As evidence on the predictors of preterm birth is limited in Nepal, we suggest additional studies to validate the findings.

There are some strengths and limitations of our study. The major strength of this study is that it employs a case-control study design. In the study, the recruitment of control immediately after the case helped to minimize the risk of selection bias. Also, we retrieved some of the clinical information from the maternity service register which helped reduce the recall bias. One of the study's limitations was that this study was conducted in a hospital in Kathmandu Valley and thus cannot be generalized to other parts of the country. Similarly, the classification of term birth and preterm birth was based on the women's recall of their LMP date, which might have affected the findings. The study does not distinguish between spontaneous and medically indicated preterm birth. The study did not assess whether the study participants had a helminthic infection. Only the administration of antihelminthic treatment was evaluated. Also, this study did not analyze other risk factors such as the mother's mental health status and other environmental factors such as heavy metal and pesticides.

## Conclusion

This study found that preterm birth was protective in women who had consumed antihelminthic drugs as per ANC protocol. Exposure to indoor air pollution and intense physical activity during pregnancy were also significantly associated with preterm birth. Based on the study findings, we recommend that maternal health programs consider factors such as counseling pregnant women for antihelminthic consumption, taking rest during pregnancy, and reducing exposure to indoor pollutants. Further prospective cohort studies are needed in the future to identify the causes of preterm birth.

## Data Availability Statement

The raw data supporting the conclusions of this article will be made available by the authors, without undue reservation.

## Ethics Statement

The studies involving human participants were reviewed and approved by Institutional Review Board, Tribhuwan University Teaching Hospital. The patients/participants provided their written informed consent to participate in this study.

## Author Contributions

RA contributed to the design and implementation of the research, to the analysis of the results, and to the writing of the manuscript. PK contributed to the analysis of data and writing the manuscript. HB contributed to the analysis of data, writing the manuscript, and review of the article. AA contributed to the overall supervision of the research and review of the article. All authors contributed to the article and approved the submitted version.

## Conflict of Interest

The authors declare that the research was conducted in the absence of any commercial or financial relationships that could be construed as a potential conflict of interest.

## Publisher's Note

All claims expressed in this article are solely those of the authors and do not necessarily represent those of their affiliated organizations, or those of the publisher, the editors and the reviewers. Any product that may be evaluated in this article, or claim that may be made by its manufacturer, is not guaranteed or endorsed by the publisher.

## Abbreviations

ANC: antenatal care
AOR: adjusted odds ratio
BMI: body mass index
CI: confidence interval
C/S: cesarean section
ICU: intensive care unit
NDHS: Nepal Demographic Health Survey
OR: odds ratio
PCA: principal component analysis
PTB: preterm birth
RR: relative risk
SPSS: Statistical Package for Social Sciences
TD: tetanus diphtheria
TT: tetanus toxoid
VIF: variance inflation factor
WHO: World Health Organization.
