# Supplementary material for: Risk Factors of Preterm Birth in Nepal: A Hospital-Based Matched Case-Control Study
Source: Front Reprod Health. 2021 Aug 30;3:697419. doi: 10.3389/frph.2021.697419 (PMC9580705; doi:10.3389/frph.2021.697419)
Supplement: Supplementary file 1 [file Data_Sheet_1.docx]

**Supplementary Material File 1**

**Operational definitions of some variables**

**Wealth Quintile:** The wealth quintile is assessed by adapting the validated assets index of NDHS. The assets included in the study were electricity, radio, television, landline phone, mobile phone, refrigerator, improved cooking stove, table, chair, bed, sofa, cupboard, computer, wall clock, fan, dhiki/jhato, microwave oven, washing machine, watch, laptop, dwelling characteristics like roof material, cooking fuel, toilet facility, number of rooms, separate kitchen and ownership of domestic animals, vehicles and land. The asset index is converted into assets scores using Principal Component Analysis (PCA). Based on asset scores, the wealth quintile are classified as the lowest wealth quintile, middle wealth quintile and the highest wealth quintile.

**Early pregnancy BMI:** It is the BMI of women calculated with weight taken before 20 weeks gestation. It is classified based on WHO criteria as: thin: < 18.5 kg/m^2^, Normal: 18.5- 24.9 kg/ m^2^ and overweight/ Obese: ≥ 25kg/ m^2^ (16).

**Anemia:** WHO cutoffs for the hemoglobin level has been adopted. According to the WHO anemia is defined as hemoglobin level less than 11 gm/dl. It is classified as mild anemia: 10-10.9; moderate anemia:7–9.9 g/dl; severe anemia:<7 g/dl (17).

**Pregnancy problems:** It is the presence of any of the following problems during pregnancy: vaginal bleeding, severe gestational hypertension, gestational hypertension, eclampsia, oligohydramnios, hepatitis B positive and urine infection.

**Exposure to second hand smoking:** It is defined as women during her pregnancy reported to have exposed to cigarettes smoking either in home or workplace.
